# Supplementary material for: Characteristics of Distinct Dietary Patterns in Rural Bangladesh: Nutrient Adequacy and Vulnerability to Shocks
Source: Nutrients. 2021 Jun 15;13(6):2049. doi: 10.3390/nu13062049 (PMC8232582; doi:10.3390/nu13062049)
Supplement: Supplementary file 1 [file nutrients-13-02049-s001.zip › nutrients-1263815-supplementary.pdf]

Table S1: 32 food groups from 127 foods data

No.	Food group	Component foods
1.	Cereal-rice	Rice, fine rice, rice flour, suji
2.	Cereal-wheat	Wheat, chira, atta/moida,
3.	Cereal-other	Noodles, semai, other cereal
4.	Pulse-lentil	Lentil
5.	Pulse-blackgram	Black gram
6.	Pulse-other	Khesari, Mashkalai, mung, chick pea, other pulse
7.	Vegetable-oil	Soyabean/palm oil, mustard/sesame oil, ghee, other
8.	Vegetable-egg plant	Egg plant
9.	Vegetable-tomato	Tomato
10.	Vegetable-gourd	Ash gourd, sweet gourd, jhinga, bitter gourd
11.	Vegetable-cabbage	Cabbage, cauliflower
12.	Vegetable-bean	Long bean, soybean bori
13.	Vegetable-onion	Onion, garlic
14.	Vegetable-radish	Radish
15.	Vegetable-other	Patal, cucumber, okra, drumstick, green banana, papaya, katchu, danta, carrot, green chilli, jhinga, other vegetable
16.	Potato	Potato
17.	Leafy vegetable-pui	Pui
18.	Leafy vegetable-other	Kalmi, lal shak, bathua, lau shak, kachu, blackgram leaves, mustard leaves, radish leaves, onion/garlic stalk, pat shak, mixed leafy vegetables, tea leaf, other leafy vegetable
19.	Chicken	Chicken
20.	Beef	Beef
21.	Meat-other	Mutton/goat, pork
22.	Egg	Eggs
23.	Fruit-jujube	Jujube/olive
24.	Fruit-banana	Banana
25.	Fruit-orange	Orange
26.	Fruit-apple	Apple, bell/wood apple
27.	Fruit-other	Tamarind, jaamrul, chalta, ata, guava, papaya, dalim, grapes, coconut, lemon, other fruit
28.	Fish-large	Hilsa, grass carp, boal, should, tilapia, singi, kalibaus, baim, tortoise, dry fish, other big fish
29.	Fish-small	Puti, moa, pabda, koi, tatkeni, prawn, other small fish
30.	Dairy	Cow/goat/sheep milk, condensed milk, powder milk
31.	Sweets	Sugar, sweets/curd, biscuits/cookies, prepared tea, soft drink/coke, pack juice
32.	Spices	Dried chilli, tejpata, turmeric, corianda, jira, elachi, panchforan, ginger, salt, other spice

S1: Latent class analysis modelling

We ran latent class models for both continuous and categorical variables and chose outputs with better model fit characteristics and showed more local relevance. With increasing number of classes from one to seven, we identified the best class solution for the data. Random iteration starts were specified at 50 to 2000 increasing with increasing number of classes specified in LCA. We stopped at a maximum of seven classes because there was no improvement in model fit statistics and convergence problems emerged despite increasing the number of random starts. Goodness of fit statistics of the models considered for choosing the best class solution for the data were Akaike information criterion (AIC), Bayesian information criterion (BIC) and entropy of class membership [(1)]. Models with the lowest AIC and BIC, and a high entropy had best fit to the data and were selected for the regression modelling. In addition to model fit statistics, we considered interpretability of the solutions in choosing the final class solution for the data.

Table S2: Latent class model fit statistics

Number of classes	AIC	BIC	Entropy	Probability of membership
1	68850.72	69150.54	1	1:0
2	67319.96	67774.37	1	0.30:0.70
3	65768.14	66377.14	1	0.030:0.68:0.29
**4**	**64261.56**	**65025.15**	**1**	**0.33:0.33:0.32:0.02**
5	63498.16	64416.34	1	0.02:0.33:0.32:0.30:0.03
6	62999.22	64072	0.939	0.02:040:0.15:0.32:0.08:0.03
7	62354.91	63582.28	0.945	0.02:0.39:0.32:0.15:0.02:0.07:0.03

S2: Statistical modelling Strategy

We used the likelihood ratio test to assess the suitability of a 3-level model which includes both households and districts as random effects or a 2-level model which includes only households as random effects as best fit for the data. The most suitable model level was chosen for the data.

The decision to include a covariate in the multivariable models was based on theorised causal relationships between variables and informed by best model fit statistics using the likelihood ratio test. We first tested for bivariate associations between explanatory variables and the outcomes using chi-squared tests. All explanatory variables were either binary or categorical in nature. We performed tests of departures from linearity to assess the suitability to use either a linear or categorical form of explanatory variables in the models which also used the likelihood ratio test. Reference categories for explanatory binary/categorical variables in our models were those with more participants. Where this rule made interpretation difficult, we chose a reference group that improved interpretation of the results. We assessed the possibility of multi-collinearity among the fixed effects in our models by checking changes in standard errors when other variables were added to the models. Interactions among explanatory variables in the models were also checked by fitting interaction terms between covariates of interest.

Dietary patterns with fewer observations faced data sparsity problems in mixed effects models. This meant that the models did not have enough outcome data to estimate multiple odds ratios. For this reason, mixed effects models were not fitted. Instead, we used univariate logistic regression to explore unadjusted associations between explanatory variables.

1. Patterson BH, Dayton CM Graubard BI (2002) Latent class analysis of complex sample survey data: application to dietary data. *Journal of the American Statistical Association* 97, 721-741.

Table S3: 32 food groups and mean individual daily consumption (in kcal) by identified dietary patterns

No.	Food group	Rice & low diversity (mean)	Wheat & high diversity (mean)	Pulses & vegetables (mean)	Meat & fish (mean)
1.	Cereal-rice	2403.08	1968.38	2248.05	2533.96
2.	Cereal-wheat	32.06	208.64	77.64	108.92
3.	Cereal-other	0.23	3.09	0.57	0.10
4.	Pulse-lentil	13.20	32.40	18.35	48.12
5.	Pulse-blackgram	8.06	16.18	14.61	0.10
6.	Pulse-other	6.31	21.12	11.15	0.31
7.	Vegetable-oil	118.39	239.66	164.43	170.85
8.	Vegetable-egg plant	7.67	12.10	9.71	10.18
9.	Vegetable-tomato	1.36	2.96	1.88	1.89
10.	Vegetable-gourd	0.95	2.50	1.43	0.81
11.	Vegetable-cabbage	12.05	18.52	14.30	13.96
12.	Vegetable-bean	12.48	28.79	19.58	23.41
13.	Vegetable-onion	12.01	21.34	15.73	15.61
14.	Vegetable-radish	4.65	5.20	5.24	1.88
15.	Vegetable-other	31.25	45.67	37.81	30.14
16.	Potato	50.18	77.31	67.78	67.17
17.	Leafy vegetable-pui	2.29	4.81	2.77	4.44
18.	Leafy vegetable-other	11.19	19.19	15.77	10.26
19.	Chicken	15.08	3.95	24.82	24.99
20.	Beef	1.96	6.93	3.42	4.18
21.	Meat-other	0.14	0.16	0.22	38.11
22.	Egg	0.47	1.10	0.84	0.733
23.	Fruit-jujube	8.32	21.79	14.77	5.50
24.	Fruit-banana	0.25	0.59	0.34	0.33
25.	Fruit-orange	0.10	0.99	0.58	0.56
26.	Fruit-apple	1.05	5.18	1.60	4.64
**27.**	Fruit-other	1.98	4.92	2.03	2.89
28.	Fish-large	37.50	96.76	57.58	79.03
29.	Fish-small	12.50	25.65	17.71	18.50
30.	Dairy	2.29	16.80	3.67	13.28
31.	Sweets	34.23	103.92	66.95	82.30
32.	Spices	16.64	30.96	21.73	24.55

Table S4: Distribution of proportions meeting WHO recommendations by selected sample characteristics

Characteristic	Proportion meeting WHO recommendations			
	Carbohydrate (%)	Fat (%)	Protein (%)	Fruit and vegetables (%)
**Household wealth**				
Poor	100.0	15.0	21.3	17.2
Medium	97.5	20.6	28.1	17.5
Rich	99.4	24.7	28.1	20.0
**Educational level[a]**				
None	99.7	16.3	23.1	15.0
Primary school	98.5	20.9	25.7	19.5
Secondary/Tertiary	100.0	33.8	35.0	30.0
**Household size**				
1-4	98.9	30.8	25.7	27.0
5-7	99.7	11.8	25.4	11.0
>7	98.7	5.0	23.7	10.0
**Region of residence**				
Northern	99.0	23.5	15.0	17.5
Eastern	99.3	18.0	31.7	19.0
Central	99.3	16.4	25.0	24.3
Southern	99.4	22.5	26.9	13.1

[a] Characteristic of household head

Table S5: Univariable predictors of dietary pattern in Bangladesh

Predictor	Rice & low diversity (n=263)		Wheat & high diversity (n=262)		Pulses & vegetables (n=259)	
	OR (95%CI)	p-value	OR (95%CI)	p-value	AOR† (95%CI)	p-value
**Household wealth**						
Poor	1	<0.001	1	<0.001	1	0.50
Medium	0.38 (0.25-0.59)		2.24 (1.48-3.40)		1.23 (0.83-1.84)	
Rich	0.38 (0.27-0.53)		2.55 (1.80-3.61)		0.99 (0.71-1.38)	
**Age group (years)**						
≤35	1	<0.001	1	<0.001	1	0.24
36-45	2.34 (1.56-3.51)		0.48 (0.32-0.71)		0.91 (0.60-1.36)	
46-55	1.40 (0.90-2.18)		0.55 (0.36-0.82)		1.28 (0.84-1.94)	
>55	1.73 (1.12-2.69)		0.46 (0.30-0.71)		1.30 (0.85-1.98)	
**Religion**						
Muslim	1	0.002	1	0.02	1	0.96
Other	0.41 (0.23-0.72)		1.71 (1.1-2.67)		1.01 (0.63-1.62)	
**Educational status**						
None	1	<0.001	1	<0.001	1	0.80
Primary school	0.57 (0.42-0.78)		1.56 (1.13-2.14)		1.10 (0.80-1.51)	

Secondary/Tertiary	0.23 (0.11-0.45)		2.35 (1.43-3.86)		1.12 (0.67-1.87)	
**Household size**		<0.001		<0.001		0.79
1-4	1		1		1	
5-7	2.07 (1.50-2.86)		0.45 (0.33-0.62)		1.08 (0.79-1.48)	
>7	2.90 (1.78-4.77)		0.36 (0.21-0.65)		0.92 (0.54-1.55)	
**Farm production**		0.03		0.03		0.97
<5	1		1		1	
6-10	0.74 (0.531.04)		1.35 (0.95-1.93)		0.96 (0.68-1.36)	
>10	0.56(0.36-0.88)		1.78 (1.16-2.74)		0.96 (0.62-1.48)	
**Region of residence**		0.007		0.03		0.72
Northern	2.05 (1.31-3.24)		0.51 (0.33-0.80)		0.82 (0.53-1.28)	
Eastern	1.45 (0.94-2.22)		0.76 (0.51-1.13)		0.94 (0.62-1.40)	
Central	1.12 (0.687-1.87)		0.86 (0.53-1.37)		1.06 (0.66-1.70)	
Southern	1		1		1	
